# Health Behavior and Lifestyle Trends among Platelet Donors: Results from a Questionnaire-Based Survey in Norway

**DOI:** 10.1155/2021/8891885

**Published:** 2021-03-22

**Authors:** Seyed Ali Mousavi, Brita Hermundstad, Per Christian Saether, Monica Jenssen Nybruket, Teresa Risopatron Knutsen, Abid Hussain Llohn

**Affiliations:** ^1^Department of Immunology and Transfusion Medicine, Akershus University Hospital, Norway; ^2^Department of Multidisciplinary Laboratory Medicine and Medical Biochemistry, Akershus University Hospital, Norway

## Abstract

**Background:**

Blood donors are on average healthier than the general population, a phenomenon known as the “healthy donor effect.” Earlier studies have also pointed to healthier behaviors among whole blood donors than the general population. This study is aimed at assessing the prevalence of four healthy behaviors (sufficient physical activity, avoiding cigarette smoking, low to moderate alcohol use, and maintaining a healthy weight) among platelet donors and to compare the results with those in the general population of similar ages.

**Methods:**

Eighty-six platelet donors were asked to complete a questionnaire designed to assess physical activity, smoking, and alcohol use. Sociodemographic information including gender, age, and education was also collected from all participants. Chi-square statistics and logistic regression were used in statistical analysis.

**Results:**

The mean age of the study donors was 51 years, 56% were female. Most were employed (90%), and 48% hold a bachelor's or higher degree. The prevalence of healthy behaviors differed by education gradients but not by gender and age. About 49% of the donors met the weekly physical activity recommendations, less than 5% were daily smokers, and~26% were classified as more frequent drinkers (≥1 to ≤5 times per week). The corresponding percentages for the general population were, respectively, 33%, 13%, and 35%. The prevalence of overweight and obesity, as assessed by body mass index (BMI), among donors were 50% and 29%, respectively, much higher than the current prevalence of overweight and obesity of 37% and 19%, respectively, among adults in the general population.

**Conclusions:**

The individual health behaviors of the majority of the study population could be characterized by a relatively high level of physical activity, low prevalence of daily smoking, and moderate alcohol drinking. The above-average overweight/obesity prevalence among platelet donors in this cohort is of concern because of the potential serious health consequences and it warrants further reflection.

## 1. Introduction

Several decades ago, Sullivan [1] proposed that the decreased risk of cardiovascular diseases (CVD) in premenopausal women, as compared to postmenopausal women and age-matched men, could be attributed to lower levels of iron and serum ferritin due to menstrual bleeding. Sullivan suggested that the depletion of iron stores with repeated blood donation in postmenopausal women and men might also protect against CVD. In support of this “iron hypothesis,” early prospective studies comparing whole blood donors with nondonors reported an association between blood donation and reduced risk for CVD [2, 3].

More recent studies have also suggested that blood donation may confer cardiovascular and metabolic benefits for blood donors. For example, an observational study among German blood donors suggested that regular blood donation is associated with a marked reduction in systolic and diastolic blood pressures among hypertensive donors [4]. However, a study among American blood donors suggested that regression to the mean may have contributed to these positive results [5]. In another recent study conducted at two Mediterranean blood banks in Italy and Greece, it was observed that regular blood donation in Greek blood donors could positively affect total oxidative status, a measure of overall antioxidant capacity, as reflected in enhanced activity of antioxidant enzymes in serum [6].

The association between blood donation and potential health benefits, including a protective effect against CVD, may, however, be complicated by the phenomenon called the “healthy donor effect” (HDE). This is akin to the concept of the “healthy worker effect” in occupational cohort studies, whereby individuals who apply and enter the industry are healthier and have lower morbidity and mortality than the general population [7, 8]. The HDE is the selection bias due to donor eligibility criteria which select for individuals healthy enough to donate blood, and volunteer bias, because healthier individuals may be more likely to choose to become blood donor [9, 10]. The HDE poses therefore a problem when comparing blood donors with the general population, because blood donors are on average healthier than the general population, making the general population an inappropriate comparison group. Findings of a recent large study comparing two indicators of health, self-rated physical health status, and mental health status, between Danish blood donors with nondonors, which showed that blood donors had better-reported health than the comparison group members are consistent with the concept that blood donors are healthier than the general population [11].

Two recent studies have tried to circumvent this problem by using internal comparison groups. In an analysis of nearly 160 000 Dutch, whole blood donors with a history of at least 10 years of active donation in which high-frequency donors were compared with low-frequency donors, a study found out that high-frequency female, but not male, donors, had a 9% decreased risk for cardiovascular morbidity compared with low-frequency female donors (age-adjusted hazard rate ratio: 0.91, 95% CI: 0.85-0.98) [12]. Moreover, sensitivity analyses repeated with a 5-year qualification period yielded similar results, supporting the absence of a residual HDE. In another large study which included almost 1.2 million whole blood donors aged 18-64 years in Denmark and Sweden, current donors were compared with donors who had stopped donating blood due to advanced age. It was found that blood donation was positively related to greater life expectancy [13], suggesting that part of this gain may be due to the effects of blood donation. Shehu and colleagues [14] attempted to quantify the magnitude of the HDE among German blood donors and found that a large part (~82%) of the observed differences in health status between donors and nondonors could be explained by the HDE.

There is also evidence that the magnitude (or strength) of the HDE is influenced by a number of factors, including sociodemographic and lifestyle variables. For instance, in a study of Dutch blood donors, researchers found that whole blood and plasma donors were more educated, were less likely to smoke, had a lower prevalence of alcohol consumption, had more self-reported physical activity, and were slightly more likely to engage in healthful food choices than the general population. Furthermore, compared to the general population, they had fewer reported health conditions, including a lower prevalence of type 2 diabetes and high cholesterol, had fewer recent (past three months) doctor visits, and were less likely to be treated at a specialist's office during the past six months [10, 15]. Similarly, German blood donors were less likely to smoke and more likely to consume healthy diets and had a lower prevalence of overweight and lower prevalence of chronic diseases than nondonors and inactive donors [14]. These data suggest that whole blood and plasma donors may have healthier behaviors than the general population.

Platelet donors represent a unique population of volunteer, unpaid blood donors because of the high demand for platelet concentrates to support transfusion therapy. Regular platelet donors are willing to give this life-saving gift every time they are able to donate and spend extra time, which is necessary for an apheresis platelet collection. However, studies published until now have not assessed health behaviors among platelet donors. This study is aimed (1) at assessing the prevalence of four positive health behaviors (i.e., sufficient physical activity (PA), avoiding cigarette smoking, low to moderate alcohol use, and maintaining a healthy weight) in a representative sample of platelet donors, (2) at examining how these healthy behaviors varied according to sociodemographic factors, and (3) at comparing these results with those reported in nationally representative surveys that include both men and women of similar age range.

## 2. Methods

### 2.1. Setting and Study Population

The study was conducted at the Blood bank in Akershus University Hospital in Loerenskog, Norway, between April 4, 2019, and February 18, 2020. The study was approved by the local Institutional Review Board, and all participants provided written informed consent. Eighty-six apheresis platelet donors attending their donation appointment were asked to participate in the study by completing a questionnaire. There were no exclusion criteria for donors, other than being ineligible to donate platelets. The questionnaire was designed to collect data on donor sociodemographics (gender, age, relationship status, education level, employment status, weight, and height) and health behaviors (physical activity, cigarette smoking, and alcohol use). Each questionnaire was given a unique research identification number to guarantee anonymity. The questionnaire was pilot-tested among five donors and based on comments from donors and interviewing/apheresis staff; questions were modified to improve clarity. The questionnaire took 10-15 minutes to complete. Self-reported weight and height were cross-checked with the collection protocol data and used to calculate the body mass index (BMI) and weight in kilograms divided by height in meters squared (kg/m^2^). Educational attainment was assessed by the highest self-reported grade completed and categorized into four education levels: middle school (at least 9 years of education completed), high school degree or equivalent (12 years of education completed), bachelor's degree (had completed 3-4 years of education beyond high school), and higher degree (had completed 5 or more years of education beyond high school).

### 2.2. Questions on Self-Reported Health Behaviors

#### 2.2.1. Physical Activity

Frequency and duration of PA question asked “How often during leisure time are you engaged in at least 30 minutes physical activity?” The question had 6 responses of (1) never, (2) sometimes, (3) once per week, (4) 2 times per week, (5) 3-4 times per week, and (6) 5-7 times per week. The participants were then asked to state their PA intensity levels as light intensity (breathing approximately normal, equivalent in effort to slow walking/leisurely walk), (2) moderate intensity (activities that cause light sweating or slight to moderate increases in breathing, equivalent in effort to brisk walking), or (3) vigorous intensity (activities that cause heavy sweating or breathing much harder than normal, equivalent in effort to running, jogging and bicycling). In addition, they were asked whether they were engaged in training in gyms or fitness centers/studios (yes/no) and then whether they have had physical activity in the past 24 hours (yes/no).

#### 2.2.2. Smoking

For smoking habits, participants were asked “Do you smoke?” with the following response options: never smoked, former smokers, daily smokers, and occasional smokers. Those who smoked were further asked whether they had smoked in the past 24 hours (yes/no).

#### 2.2.3. Alcohol Consumption

The frequency of alcohol consumption question asked “How often do you drink alcohol?” In all, eight response options were available: (1) never, (2) less than once per month, (3) once per month, (4) 2-3 times per month, (5) once per week, (6) 2-3 times per week, (7) 4-5 times per week, and (8) every day or almost every day. The participants were then asked: “How many drinks they consume on a typical drinking occasion?” This question was answered using a 4-point scale with response options ranging from 1 to 4+ drinks per occasion. Study participants were also asked whether they had consumed alcohol in the past 24 hours (yes/no).

#### 2.2.4. Self-Reported Sleep Measures

The questionnaire also included a single-item measure of subjective sleep quality in which donors were asked to rate their sleep quality on a five-point scale from very good to very poor. In addition, donors were asked about their habitual bedtimes and duration of sleep.

#### 2.2.5. Engagement in Multiple Healthy Behaviors

The number of positive health behaviors (i.e., sufficient PA, avoiding cigarette smoking, low to moderate alcohol consumption, and maintaining a healthy weight) was summed for each donor. Donors were categorized as having 0, 1, 2, 3, or 4 positive health behaviors.

### 2.3. Statistical Analysis

For the purpose of our analysis, sociodemographic and health behavioral variables were mostly dichotomized: gender (male, female), age (22-50 vs. 51-69 years), relationship status (married/cohabiting vs. single), and education level (high school or less vs. bachelor's or higher degree). Participants were also classified as employed if they were in paid employment; otherwise, classified as “other”. Sleep quality was dichotomized as very good/good vs. fair. Smoking status was categorized into nonsmokers (never smokers + former smokers) and current smokers (daily smokers + occasional smokers). An indicator of PA levels per week was calculated as the product of frequency and intensity of PA reported by each donor. For current drinkers, we calculated the average “typical” number of drinks consumed per month by combining frequency and quantity (i.e., the product of frequency × quantity) into a single continuous variable. For comparison with the general population, current drinkers were also categorized as less frequent drinkers (≤3 times per month) or more frequent drinkers (≥1 to ≤5 times per week), because no equivalent data were available in the most recently published statistics for alcohol consumption. Finally, for bivariate comparison of the number of positive health behaviors, a dichotomized variable was produced by combining the scores 1 and 2 and scores 3 and 4. Because the majority of participants were employed and married/cohabiting and rated their sleep quality as very good/good, differences in these variables could not be evaluated.

Data were summarized with descriptive statistics and expressed as number and percentage/proportion for categorical variables and mean ± standard deviation (SD) for continuous variables. Cross-tabulation, a chi-square test, or the Fisher's exact test were used to analyze differences between groups. Multivariate logistic regressions were used to estimate adjusted odds ratios (OR) and associated 95% confidence intervals (CI). Independent samples *t*-test was used to compare differences in means of continuous variables and the Pearson correlation coefficient (Pearson's *r*) to evaluate their correlations. Statistical testing was 2-sided, with *p* < 0.05. Analyses were performed with IBM SPSS Statistics, version 25. The data set did not have any missing values.

## 3. Results

### 3.1. Sociodemographic and Health Behavioral Characteristics of the Study Population

We asked 86 donors (~80% of the platelet donor pool at our blood bank) to participate in the study. All of the donors asked to participate agreed to do so and completed the questionnaire. The number and percentage distribution of responses to questionnaire items are shown in Table 1. The mean age was 50.8 ± 9.7 years (range: 22-69 years), 55.8% were female, and 78% were married or cohabiting (53.5% and 24.5%, respectively). Most (89.5%) were employed; the remainder were recently retired (5.8%) or students (2.3%). Only one donor was presently unemployed and only a 47-year-old donor reported receiving a temporary disability benefit. Slightly more than half of the donors had high school or less education (46.5% and 5.8%, respectively), and 47.7% hold a bachelor's or higher degree (33.7% and 14%, respectively). Regarding ethnicity, only one donor had an ethnic minority background. Male and female donors did not differ in age (52.1 ± 8.7 vs. 49.5 ± 10.4 years, *p* = 0.22), but more males (61%) than females (38%) hold a bachelor's or higher degree (*p* = 0.034).

### 3.2. Sleep Quality and Quantity

Regarding sleep quality, approximately 85% rated their sleep quality as very good or good and 15% rated their sleep as fair. None of the donors rated their sleep as poor or very poor. The average reported sleep duration per night was 6.8 ± 0.8 hours (range: 4.5 to 9.0 hours). Two donors reported less regular sleep schedules due to undertaking shift work. Sleep quality was neither significantly associated with sociodemographic nor with health behavior variables.

### 3.3. Health Behavior Characteristics

Table 2 summarizes the prevalence of meeting weekly PA recommendations, gym use, donors who had performed PA in the past 24 hours, smoking status, and drinking status according to gender, age group, and education level. The results of the analysis of the number of positive health behaviors are also presented in Table 2.

#### 3.3.1. Leisure-Time Physical Activity Behavior

In terms of PA frequency, 11.6% of donors reported to be physically active less than once per week, 5.8% reported at least once per week, 18.6% two times per week, 29.1% three to four times per week, and 34.9% five times or more per week (Table 1). No study participants reported not doing PA at all. Of the self-reported physical activity, 16.3% was light-intensity activity, 46.5% was moderate-intensity activity, and 37.2% was vigorous-intensity activity (Table 1). The proportion of donors who reported training in gyms or fitness centers/studios (hereafter referred to as gym users) was 44.2%, and the same percentage had performed PA in the past 24 hours (Table 2).

Gym use did not vary by gender and age, but a slightly higher percentage of donors with higher education than those with lower education reported gym use (54% compared to 36%, *p* = 0.091). About 57% of older donors had performed PA in the past 24 hours compared with 31% among their younger counterparts (*p* = 0.016). A significantly higher percentage of donors with higher education than those with lower education had also performed PA in the past 24 hours (51% vs. 38%, *p* = 0.021). Among those who reported gym use, about 50% reported having PA during the past 24 hours compared with 40% among those who did not use gym (unadjusted OR: 1.53, 95% CI: 0.65-3.61, *p* = 0.32, not shown in Table 2).

Analysis of PA levels per week showed that donors' activity levels fell within three groups: (1) those with at least 150 min (20.9%) or at least 90 min (11.6%) of vigorous activity per week, (2) those with at least 150 min of moderate activity per week (16.3%), and (3) those with PA at the levels which were below 150 min moderate or below 75 min vigorous activity per week (51.2%). The latter group comprised those with at least 90 min of moderate activity per week (12.8%) and those with activity levels ranging from 30 to 150 min light activity per week (38.4%). These results indicate that approximately 49% (i.e., group 1 + group 2) of the study participants met weekly PA recommendations, defined, according to national physical activity guidelines for adults [16], as at least 150 min per week of moderate-intensity activity, or at least 75 min per week of vigorous-intensity activity, or an equivalent combination, whereas 51% (i.e., group 3) failed to meet the minimum recommended activity level. Interestingly, among those who had performed PA in the past 24 hours, the average daily activity was 91 ± 82 min (range: 60 to 420 minutes). These donors seem to meet the higher recommended weekly target (≥300 minutes/week) and could therefore be considered as highly active.

Male and female donors engaged in the same level of PA (Table 2). There was no significant difference in meeting weekly PA recommendations by age group (Table 2). A significantly higher percentage (61%) of donors with higher education met weekly PA recommendations than did those with lower education (39%). Gym users and those who had performed PA in the past 24 hours had also much higher percentages (74% and 63%, respectively) that met the weekly PA recommendations (see Table 3).

A multiple logistic regression analysis that assessed these (unadjusted) associations of gym use, PA in the past 24 hours, and education with meeting weekly PA recommendations in the same model (Table 3) showed that mutual adjusting had a minimal impact on the association between gym use and PA in the past 24 hours with meeting weekly PA recommendations (i.e., odds ratios changed little), but education was no longer significant. Also, gym use was the strongest single predictor of meeting weekly PA recommendations; donors who reported gym use were 6.9 times more likely to meet weekly PA recommendations than those who did not report gym use. The model explained 30% of the variance in meeting PA recommendations (Pseudo *R*^2^ = 0.303).

#### 3.3.2. Smoking Habits

Over 87% of donors reported that they had never smoked (58.1%) or were former smokers (29.1%). Only 4.7% of donors reported being daily smokers, whilst 8.1% described themselves as occasional/social smokers (Table 1). Male and female donors had a similar prevalence of daily smoking (5.2%, [2/38]; among males compared with 4.2%, [2/48]; among females), but slightly more males than females reported occasional smoking (10.5%, [4/38]; vs. 6.2%, [3/48]; among females). A higher percentage of donors in the older group were daily smokers (6.8%, [3/44]; compared with 2.4%, [1/42]; among 22-50 year-olds), but nearly the same percentage of older and younger donors reported occasional smoking (9.1%, [4/44]; vs. 7.1%, [3/42]). Only those with lower education reported daily smoking (8.9%, [4/45]; vs. 0% among those with higher education), but the proportion of occasional smokers was slightly higher among those with higher education (9.8%, [4/41]; vs. 6.6%, [3/45]; among those with lower education).

#### 3.3.3. Frequency and Quantity of Alcohol Consumption

Three donors (3.5%) described themselves as never drinkers. Among current drinkers (*n* = 83), 22.9% consumed alcohol less than once per month, 19.3% once per month, and 31.3% 2-3 times per month. The percentages of those drinking once per week, 2-3 times per week, and 4-5 times per week were 4.8%, 18.1%, and 3.6%, respectively (Table 1). No study participants reported daily or almost daily drinking. The distribution of typical number of drinks consumed per occasion was as follows: 12% drank one drink, 41% 2 drinks, 32.5% 3 drinks, and 14.5% 4+ drinks (Table 1). There was no correlation between frequency and quantity of alcohol consumption (*r* = 0.004, *p* = 0.97).

Based on drinking frequency data, 74% of the current drinkers were classified as less frequent drinkers and 26% as more frequent drinkers (Table 2). A slightly higher percentage of female (80%) than male donors (65%) and a higher percentage of younger donors (83%, aged 22-50 years) than older donors (65%, age 51-69 years) were less frequent drinkers. Donors with lower education also tended to drink less frequently than those with higher education (81% vs. 65%).

Total monthly alcohol consumption correlated significantly with both frequency (*r* = 0.85, *p* < 0.001) and quantity of consumption (*r* = 0.25, *p* = 0.025). This analysis (Table 2) showed that on the days they drink alcohol during a month, study participants consume on average 10.7 ± 12.6 drinks (median = 6, range: 1–48). About 74% (65% males and 82% females, respectively) drank 10 drinks or fewer in a month. There was no significant gender difference in average monthly alcohol consumption (males: 12.9 ± 12.5 [median = 9, range: 1–48]; females 8.8 ± 12.4 [median = 3, range: 1–48]; *p* = 0.14). Older donors consumed more alcohol than did younger donors. Donors with higher education had also higher average monthly consumption than those with lower education (Table 2). However, these differences did not reach significance. However, donors who had reported recent (past 24 hours) drinking (*n* = 9) had significantly higher average monthly alcohol consumption than those who did not report recent drinking (26 ± 16 vs. 8.8 ± 10.8, *p* < 0.001, not shown in Table 2).

Further analysis of these data revealed that the majority (92%) of our study population adhere to the guideline of having no more than 14 standard drinks for men and 7 drinks for women per week. Only 5.4% (2/38) of males and 10.4% of females (5/48) exceeded the guideline (i.e., the recommended maximum number of drinks per week). It should be noted, however, that it is not possible to calculate the maximum number of drinks consumed per occasion based on these data alone, as the item about the number of drinks consumed per occasion was limited to 4+ drinks. Thus, we do not know whether these donors were engaged in heavy or binge drinking or not.

This analysis also yielded a range of “typical” consumption patterns. For example, of those who reported to drink less than once per month (i.e., the lowest drinking frequency reported in this population, *n* = 19), 16% (*n* = 3) drank 4+ drinks per drinking occasion, whereas those reporting to drink 4-5 times per week (i.e., the highest frequency of drinking in this population, *n* = 3) drank 2 drinks per drinking occasion. In other words, frequency and quantity of consumption tended to point in opposite directions.

#### 3.3.4. Prevalence of Overweight and Obesity

The mean body weight of the study population was 85.7 ± 17.2 kg (median = 84.5, range: 51–150), and their mean height was 1.74 ± 0.09 meter (median = 1.74, range: 1.52–1.90). As expected, male donors were taller (M: 1.82 ± 0.05 [median = 1.81, range 1.69–1.90]; F: 1.68 ± 0.06 [median = 1.69, range: 1.52–1.84]) and had greater body weight (M: 93.4 ± 15.4 [median = 90, range: 71–150]; F: 79.6 ± 16.2 [median = 80, range: 51–140]) than female donors (*p* < 0.001 for both). The overall mean BMI was 28.1 ± 4.7 kg/m^2^ (median = 27.3, range 19.6–44.2).

Table 4 shows the prevalence of donors in each BMI category stratified by sociodemographic and health behavioral characteristics. About 21% were “normal weight” with a mean BMI of 22.7 ± 1.81 (range: 19.6–24.8), 50% were “overweight” with a mean BMI of 27.0 ± 1.27 (range: 25.0–29.8), and 29% were “obese” with a mean BMI of 33.8 ± 3.80 (range: 30.0–44.2), of these 8% (2/25) were morbidly obese (defined as BMI ≥ 40.0 kg/m^2^). No donor was classified as underweight (defined as BMI < 18.5 kg/m^2^). Examining sociodemographic differences in the prevalence of overweight and obesity indicated that (Table 4), when compared with donors in the normal BMI category, a higher percentage of males than females were overweight (61% vs. 42%; *p* = 0.06), but a slightly higher percentage of females were in the obese category. The prevalence of overweight/obesity did not differ by age, but it varied according to education level in a way that those with lower education had a higher prevalence of overweight and obesity. Body mass index varied also by PA status, with overweight and obesity being more prevalent in those who did not meet weekly PA recommendations. Differences in the prevalence of overweight and obesity between nonsmokers and current smokers were small and not statistically significant. Body mass index did not differ by drinking status.

#### 3.3.5. Engagement in Multiple Healthy Behaviors

The engagement in multiple healthy behaviors was investigated by summing the number of these behaviors for each donor (Table 2). This analysis showed that 11.6% of donors had one positive health behavior, and those with two positive behaviors represented the largest group (41.9%) followed by those with three positive behaviors (34.9%). Only a small proportion (11.6%) of donors had all of the positive health behaviors. The results of bivariate analysis (not in Table 2) showed that 50% of males and 44% of females had 3 or 4 healthy behaviors (*p* = 0.56), and slightly more older donors than their younger counterparts had 3 or 4 healthy behaviors (50% vs. 43%, *p* = 0.51). However, donors with higher education were significantly more likely to have 3 or 4 healthy behaviors than those with lower education (58% vs. 35%, unadjusted OR: 2.6, 95% CI: 1.07–6.1; *p* = 0.033).

### 3.4. Educational Achievement and Health Behaviors of the Study Population Compared with the General Population in Norway and with Previous Studies among Other Volunteer Blood Donor Groups

**Figure 1:**
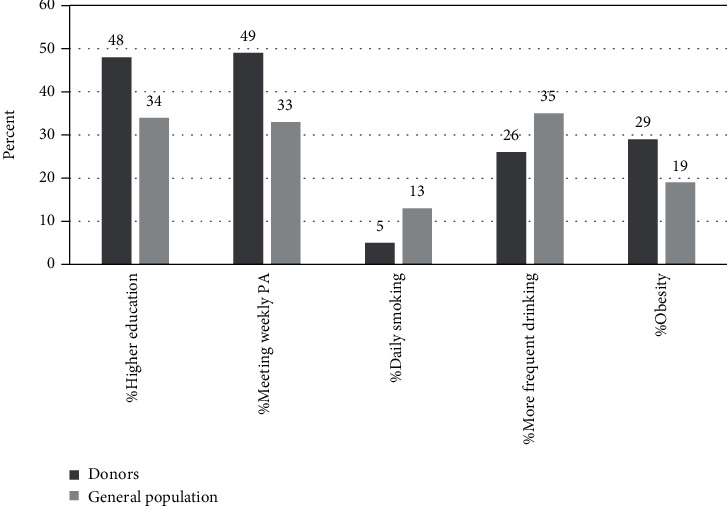
Comparison of education level and prevalence of health behaviors between platelet donors and adults in the general population (GP) of similar ages. Abbreviations: meeting PA: meeting weekly PA recommendations. Higher education is defined as bachelor's or higher degree; meeting weekly PA recommendations is defined as at least 150 min per week of moderate-intensity activity, or at least 75 min per week of vigorous-intensity activity, or an equivalent combination; more frequent drinking is defined as ≥1 to ≤5 times per week; obesity is defined as a body mass index (BMI) of >30 kg/m^2^. Source for education, daily smoking, more frequent drinking, and obesity: Norwegian Bureau of Statistics (SSB), National Survey of Living Conditions 2019. Source for meeting weekly PA recommendations: Norwegian Directorate of Health 2016.

Figure 1 shows the percentage of donors with higher education (self-reported), the prevalence of self-reported daily smoking and weekly frequency of alcohol consumption, and the calculated percentage of donors who met the weekly PA recommendations and obesity prevalence. For comparison, also displayed are the corresponding figures in the general population of similar ages. Figure 1 shows that our donor population was more educated than the national average; 48% hold a bachelor's or higher degree compared with 34% in the general population [17].

As reported by Dutch whole blood and plasma donors in previous research [10, 15], platelet donors in this study were generally more active than adults in the general population: nearly half (49%) of donors in this study met the weekly PA recommendations, substantially higher than the national figure of ~33% for 2016 [16]. It should, however, be noted that the PA assessment was based only on measures of leisure-time PA, which may not reflect their weekly total activity levels, which also include work- and domestic-related activities.

Regarding smoking habits, only <5% of donors reported daily smoking, 2.8 times lower than the rate of daily smoking (13%) in the general population (Figure 1). Approximately 8% of donors were occasional smokers, a rate close to that seen in the general population (~7.5%) in 2019 (not shown in Figure 1). These rates of smoking are still far from the ideal goal (i.e., avoidance of cigarette smoking). However, in comparison to our platelet donor population, Greek regular blood donors reported a higher rate of smoking (27.7%), similar to the prevalence of smoking among first-time donors (30%) [6]. Of note, about 29% of donors described themselves as former smokers. Among smokers in the general population in Norway, changes in smoking behavior have often focused on switching from cigarettes to “snus” (smokeless tobacco) rather than giving up smoking, perhaps because “snus” is perceived as less harmful to health than cigarettes or because snus helps them to quit smoking. It is therefore possible that some former smokers in this study are current “snus” users.

The drinking pattern of platelet donors in this study indicated that most (~74%) drank rarely (less than once per month) or were infrequent drinkers (1-3 times per month). Approximately 26% of the donors reported more-frequent drinking (consumed alcohol once per week or more), which compares favorably to the 35% rate of drinking once or more per week in the general population of similar ages (Figure 1). The majority (92%) of donors in this study tended to be light-to-moderate drinkers who consumed no more than 7 (females) or 14 (males) drinks per week (as judged by total monthly alcohol consumption). Prior research among Dutch whole blood and plasma donors found also similar results, with donors being engaged in less alcohol use when compared to the general population [10].

Finally, almost 80% of platelet donors in this study were overweight or obese, with 29.1% classified as obese. In comparison, according to 2019 data from the Norwegian National Health Survey [17], approximately 56% of adults (≥25 years) were overweight or obese, with about 19% in the obese category (see Figure 1). The prevalence of obesity among platelet donors in this study is, however, comparable to the obesity prevalence among US platelet donors (31%) in 2008 [18], but higher than the rates of obesity observed among American, Dutch, and Italian whole blood donors, 24.5%, 9.8%, and 7.7%, respectively [10, 18, 19].

## 4. Discussion

This study investigated the prevalence of four positive health behaviors among platelet donors in a blood bank in Norway. The individual health behaviors of the majority of the study population could be characterized by a relatively high level of physical activity, low prevalence of daily smoking, and moderate alcohol drinking, but high prevalence of overweight and obesity. However, only 12% of the study population had a health behavior profile that could be described as multidimensional (i.e., adhered to all four positive health behaviors examined in this study), mainly as a result of a high prevalence of overweight and obesity.

Male and female donors did not differ with regard to meeting weekly PA recommendations, daily smoking, or in terms of drinking behaviors, or the number of positive health behaviors. However, male donors had a higher prevalence of overweight than female donors, but this was only of borderline statistical significance. No significant differences in health behaviors were observed by age group, except a higher percentage of older than younger donors had performed PA in the past 24 hours. As expected, however, several educational differences were noted, with higher prevalence rates of not-meeting weekly PA recommendations, daily smoking, and overweight/obesity among those with lower education. The opposite direction was, however, found with regard to drinking frequency and average monthly alcohol consumption; those with lower education tended to drink less frequently and consumed less alcohol per month than their more educated counterparts (Table 2).

Our data suggest that platelet donors as a group appear to have healthier lifestyles than the general of similar ages: of the four health behaviors examined here, platelet donors had better health behaviors than adults in the general population in meeting weekly PA recommendations, prevalence of daily smoking, and low to moderate alcohol consumption, but not at maintaining a healthy weight. Overall, our results also appear to be in line with other studies that have compared lifestyle behaviors between other blood donor groups and the general population [10, 14, 15].

However, as blood donors are selected on their physical health, it is expected that our donor population represent a group of individuals who on average are healthier than the general population, which includes individuals with poor health and functional limitations. In other word, differences in health behaviors between our donor population and the general population may be attributable, at least in part, to the “healthy donor effect” that is operating in platelet donors, and this should be taken into account when interpreting our results. In this study, we did not ask platelet donors about their self-rated health status because donor-screening questionnaires completed by the donors before their donation indicate good general health of the donors, otherwise, they would not be eligible to donate platelets. The fact that no more than one donor (1.2%) received disability benefits, compared with 14% in the general population in Norway in 2019 [17], argues well for good health in our donor population. Further, previous research has shown that good sleep quality and sufficient sleep are positively associated with better self-rated physical and mental health [20, 21]. To the extent that good sleep quality and sufficient sleep duration reflect good health status, the majority (85%) of the study donors (see Table 1) were in good health.

Moreover, because differences in health behaviors may be due to differences in socioeconomic status, conclusions about differences in health behaviors between our donor population and the general population should also take socioeconomic differences between the two populations into account. Two indicators of socioeconomic status in our study were employment status and education level. The platelet donors in this study were characterized by high employment grade, only one (1.2%) of the donors was unemployed, compared with 6% in the general population in Norway in 2019 [17], and by higher education than the general population (48% and 34%, respectively, had a bachelor's or higher degree). For our population, the higher education resulted in donors reporting a greater number of healthy behaviors, mainly on the strength of being more likely to meet weekly PA recommendations, low prevalence of overweight and obesity, and being nondaily smokers. It is therefore possible that differences in health behaviors between our donor population and the general population reflect differences in education, as a proxy measure of socioeconomic status, to some degree.

Our finding that the prevalence of overweight and obesity is higher among platelet donors than among adults in the general population was somewhat surprising, given the fact that the overall health status in blood donors is expected to be better than the general population. This is of concern because of increased potential risks for serious health consequences from excess body weight. Platelet donors are usually recruited from experienced whole blood donors and those who donate two or three units of platelets during one donation may be required to meet the height and weight ratio for males and females to calculate donor's estimated blood volume and to guarantee minimum blood volume. Selection (donor recruitment) by blood banks for heavier whole blood donors for platelet donation may therefore favor larger (i.e., larger blood volume) whole blood donors for platelet donation. However, at our blood bank, we mainly collect a single apheresis platelet unit during one donation, and the weight criterion is the same for whole blood and platelet donors (>50 kg). Therefore, selection for heavier whole blood donors for platelet donation, as an explanation for the above-average overweight/obesity prevalence among our platelet donors, seems less likely. However, we cannot entirely rule out the possibility of a selection bias because donors may be selected according to previous platelet yield, which in turn may be related to blood volume and body weight [18].

Overweight and obesity have been linked to a wide range of lifestyle, environmental, and genetic factors. The risk of obesity has been shown to be influenced by many aspects of lifestyle including physical inactivity/sedentary behavior and unhealthy diets including energy-dense diets that have poor nutritional value such as sugar-sweetened beverages [22, 23]. In a recent cross-sectional study conducted among Greek regular blood donors in which the donors were divided into three groups of about 50 subjects based on the ratio of total cholesterol to high-density lipoprotein cholesterol (TC/HDL-C, atheromatic index), a predictor of CVD, it was found that BMI was significantly higher in those with the higher ratio of TC/HDL-C [24]. Research is needed to investigate the degree to which different plasma lipoprotein fractions contribute to total cholesterol levels among platelet donors and the TC/HDL-C ratio.

A healthy and balanced diet is a major focus of public health policies in many countries including Norway, to help maintain a healthy body weight, and reduces the risk of diet-related diseases such as CVD, cancer, and diabetes. There is evidence in the literature suggesting that frequent consumption of fruits and vegetables (low in fat and energy density) is associated with weight maintenance and decreased obesity risk [25, 26]. Results from the National Dietary Survey 2010 to 2011 among Norwegian adults aged 18 to 70 years [27] showed that adherence to fruit and vegetable intake recommendations is low: only 34% and 14% of men met fruit and berries intake recommendations (≥250 g/day) and vegetable recommendations (≥250 g/day), respectively, with corresponding values of 41% and 15%, respectively, among women.

The national survey data also indicated that alcohol intake accounted for less than 2% of total calorie intake among alcohol-consuming men and women. A generally lower level alcohol consumption in our study population and the observation that BMI did not differ among drinking groups (Table 4), as well as evidence from the nationally representative survey that alcohol consumption contributes less than 2% of energy to the diet among Norwegian adults, suggest that the energy derived from alcohol consumption may not influence body weight and BMI in this donor group. Regardless of the reasons for the differences in overweight/obesity prevalence observed between our platelet donors and the general population, our findings suggest the need to investigate the joint contribution of physical inactivity and inadequate fruit vegetable consumption to the increased weight gain and obesity among platelet donors.

However, focusing on unhealthy behaviors is potentially problematic because it raises the risk of “blaming the victim,” i.e., holding individuals personally responsible for their behavior-related health problems and because societies are also responsible to provide health-promoting environments [28, 29]. In addition, socioeconomic status has been shown to be an important predictor of obesity. In developed countries, for example, there is consistent evidence that social disadvantage increases the risk of obesity among women, although evidence for men is less consistent [30, 31]. Each of these factors may have a part to play in explaining the above-average overweight/obesity prevalence among platelet donors in this study.

### 4.1. Strengths and Limitations

This study has several strengths. First, to our knowledge, this is the first study to describe health behaviors among this population of blood donors. Second, we examined not only the frequency and intensity of PA reported by each donor but also calculated the percentage of the donor population meeting activity recommendations by gender, age, and education (Table 2). Similarly, a combined (frequency × quantity) variable was constructed in order to estimate monthly alcohol consumption levels. Finally, the potential for selective response seems unlikely because all donors who were asked consented to participate in the study (i.e., 100% response rate).

Certain limitations of this study should also be noted. First, the sample size of this study was small, which resulted in small numbers in some subgroups, particularly the relatively small number of donors in the smoking, more frequent drinking, and normal BMI categories. Lack of associations may therefore reflect limited statistical power to detect smaller (but potentially important) differences between groups. This also limits the generalizability of the study findings. However, because this study incorporated about 80% of the platelet donor pool, our population is considered representative of the Akershus blood bank. The findings of this study are, however, limited to a single blood bank, and therefore the results may not be generalizable to other blood banks/blood collection agencies. Second, physical activity, smoking, and alcohol consumption were self-reported and may therefore be subject to social desirability bias. Third, we did not collect information on dietary habits. Therefore, unfortunately, we were not able to examine the prevalence of meeting dietary guidelines and its relationship with BMI status. Fourth, self-reported health behaviors were only assessed at one point in time and thus may not reflect long-term trajectories of actual lifestyles. Finally, the study population was ethnically very homogenous as all participants except one were ethnic Norwegians, so the findings may only be generalizable to the majority population.

## 5. Conclusions

Our study assessed the prevalence of four positive health behaviors among a cohort of platelet donors, examined the role of sociodemographic characteristics in explaining differences in health behavior, and compared the results with those reported for adults in the general population. The individual health behaviors of the majority of the study population could be characterized by a relatively high level of physical activity, low prevalence of daily smoking, and moderate alcohol drinking, but high prevalence of overweight and obesity. Our findings showed that health behavior among platelet donors was most influenced by education, suggesting that factors related to socioeconomic status may affect the ability to practice healthy behaviors. Platelet donors in this study had better health behaviors than adults in the general population in daily smoking and in meeting physical activity and alcohol use recommendations, but not in maintaining a healthy weight. However, our donor population is not an entirely homogenous group with regard to health behaviors examined in this study as only 12% of donors had all four positive health behaviors, whereas 12% followed only one of these healthy behaviors. Our results also suggest that the prevalence of overweight/obesity might be disproportionately high among our platelet donors, and this is of concern because obesity not only has negative health consequences but it also may discourage healthy behaviors, particularly physical activity given the significant association between not-meeting weekly PA recommendations and obesity observed in this study (Table 4). The above-average overweight/obesity prevalence among platelet donors is most likely due to donor factors (e.g., unhealthy dietary choices and physical inactivity). Further research is required to understand the multifactorial causes of the observed differences in obesity prevalence between platelet donors and adults in the general population. This can be especially important during the current COVID-19 pandemic because it has become apparent that people with obesity, among other factors, are at increased risk of serious disease from COVID-19. For example, a systematic review and meta-analysis of 75 studies pooled data that examined the association of obesity across the full COVID-19 spectrum from risk of SARS-CoV-2 infection to mortality showed that individuals with obesity are at increased risk for infection, hospitalization, ICU admission, and mortality [32].

## Figures and Tables

**Table 1:** Characteristics of the study population (*n* = 86).

Variable	Number (%)
Age group	
22-50	42 (48.8)
51-69	44 (51.2)
Gender	
Male	38 (44.2)
Female	48 (55.8)
Relationship status	
Married/cohabiting	67 (78)
Single	19 (22)
Employment status	
Employed	77 (90)
Other^†^	9 (10)
Education level	
≤High school	45 (52)
Bachelor's or higher degree	41 (48)
Sleep quality	
Very good/good	73 (85)
Fair	13 (15)
Physical activity frequency	
<Once per week	10 (11.6)
Once per week	5 (5.8)
Twice per week	16 (18.6)
3-4 times per week	25 (29.1)
5-7 times per week	30 (34.9)
Physical activity intensity	
Light	14 (16.3)
Moderate	40 (46.5)
Vigorous	32 (37.2)
Smoking	
Never	50 (58.1)
Former smoker	25 (29.1)
Daily	4 (4.7)
Occasional	7 (8.1)
Drinking frequency^‡^	
Less than once per month	19 (22.9)
Once per month	16 (19.3)
2-3 times a month	26 (31.3)
Once per week	4 (4.7)
2-3 times per week	15 (18.1)
4-5 times per week	3 (3.6)
Typical drinks per occasion	
1	10 (11.6)
2	34 (41.0)
3	27 (32.5)
4+	12 (14.5)

Data are presented as number (percentage). Column percentages are given. ^†^Other: unemployed, retired, and students. ^‡^Only includes current drinkers.

**(a):** 

HB variables *n* (%)		Gender			Age group			Education level		
		Males	Females		22-50 yr	51-69 yr		≤High sch	≥Bachelor	
		(*n* = 38)	(*n* = 48)	*p*	(*n* = 42)	(*n* = 44)	*p*	(*n* = 45)	(*n* = 41)	*p*
PA status										
Met:	42 (49)	20 (53)	22 (46)		21 (50)	21 (48)		17 (38)	25 (61)	
Not met:	44 (51)	18 (47)	26 (54)	0.53	21 (50)	23 (52)	0.83	28 (62)	16 (39)	0.032
Gym use										
Yes:	38 (44)	18 (47)	20 (42)		20 (48)	18 (41)		16 (36)	22 (54)	
No:	48 (56)	20 (53)	28 (58)	0.60	22 (52)	26 (59)	0.53	60 (64)	19 (46)	0.091
PA last 24 h										
Yes:	38 (44)	17 (45)	21 (44)		13 (31)	25 (57)		17 (38)	21 (51)	
No:	48 (56)	21 (55)	27 (56)	0.93	29 (69)	19 (43)	0.016	28 (62)	20 (49)	0.021
Smoking status^∗^										
Nonsmoker:	75 (87)	31 (84)	44 (90)		38 (91)	37 (84)		38 (84)	37 (90)	
Current smoker:	11 (11)	6 (16)	5 (10)	0.53	4 (9)	7 (16)	0.52	7 (16)	4 (10)	0.53
Drinking status^†^										
Less frequent:	61 (74)	42 (65)	37 (80)		33 (83)	28 (65)		35 (81)	26 (65)	
More frequent:	22 (26)	13 (35)	9 (20)	0.11	7 (17)	15 (35)	0.073	8 (19)	14 (35)	0.091

**(b):** 

Average monthly use						
Mean ± SD	12.9 ± 12.5	8.8 ± 12.4	8.0 ± 11.0	13.1 ± 13.5	9.5 ± 13.1	12.0 ± 11.9
	*p* = 0.14		*p* = 0.062		*p* = 0.37	

**(c):** 

PHB scores^‡^							
1:	10 (12)	6 (16)	4 (8)	4 (9)	6 (14)	8 (18)	2 (5)
2:	36 (42)	13 (34)	23 (48)	20 (48)	16 (36)	21 (47)	15 (37)
3:	30 (35)	16 (42)	14 (29)	12 (29)	18 (41)	14 (31)	16 (39)
4:	10 (12)	3 (8)	7 (15)	6 (14)	4 (9)	2 (4)	8 (19)

Data are presented as number (percentage). Column percentages are given. Abbreviations: ≤High sch.: high school or less; ≥Bachelor's or higher degree; HB: health behavior; PA: physical activity; PHB: positive health behavior; Met: met weekly physical activity recommendations (defined as at least 150 min per week of moderate-intensity activity, or at least 75 min per week of vigorous-intensity activity, or an equivalent combination); Not met: did not met weekly physical activity recommendations. Less frequent: defined as drinking ≤3 drinks/month; More frequent: defined as drinking ≥1 ≤ 5 drinks/week. ^∗^Statistical significance used the Fisher's exact test because the number of cases was small. ^†^Only includes current drinkers. ^‡^Scores are additive with higher numbers indicating more positive health behaviors.

**Table 3:** Logistic regression predicting meeting weekly PA recommendations from the variables included in the model.

Variable	Physical activity status		Unadjusted		Adjusted†	
	Met (*n* = 42)	Not met (*n* = 44)	OR (95% CI)	*p*	OR (95% CI)	*p*
Gym use						
Yes	28 (74)	10 (26)	6.80 (2.62–17.64)	<0.001	6.88 (2.56–18.48)	<0.001
No	14 (29)	34 (71)	1.00		1.0	
PA last 24 h						
Yes	24 (63)	14 (37)	2.86 (1.18–6.89)	0.018	2.92 (1.09–7.80)	0.033
No	18 (38)	30 (62)	1.00		1.00	
Education level						
≥Bachelor's degree	25 (61)	17 (38)	2.57 (1.08–6.14)	0.032	1.99 (0.74–5.43)	0.17
≤High school	16 (39)	28 (62)	1.00			
Pseudo *R*^2^ = 0.303	*p* < 0.001					

Data are presented as number (percentage). Row percentages are given. For abbreviations: see Table 2. ^†^Adjusted OR is the odds ratio after controlling for other variables in the model.

**Table 4:** Distribution of the body mass index (BMI) categories by sociodemographic and health behavioral characteristics.

Variable	Normal	Overweight	*p*	Obese	*p*
Overall	18 (21)	43 (50)		25 (29)	
Gender					
Male	5 (13)	23 (61)		10 (26)	
Female	13 (27)	20 (42)	0.06	15 (31)	0.41
Age group					
22-50 y	7 (17)	22 (52)		13 (31)	
51-69 y	11 (25)	21 (48)	0.38	12 (27)	0.40
Education level					
≥Bachelor's degree	13 (32)	17 (41)		11 (27)	
≤High school	5 (11)	26 (58)	0.02	14 (31)	0.06
PA status					
Met	13 (31)	29 (48)		9 (21)	
Not met	5 (11)	23 (52)	0.06	16 (37)	0.02
Smoking status					
Nonsmoker	15 (20)	37 (49)		23 (31)	
Current smoker	3 (27)	6 (55)	0.78	2 (18)	0.38
Drinking status					
Less frequent	12 (20)	32 (52)		17 (28)	
More frequent	6 (27)	10 (46)	0.45	6 (27)	0.61

Data are presented as number (percentage). Row percentages are given. For abbreviations: see Table 2.

## Data Availability

All relevant data are included in the manuscript but are available from the corresponding author on reasonable request.

## Abbreviations

CVD: Cardiovascular disease
HDE: Healthy donor effect
PA: Physical activity.
